# A case report of autoimmune glial fibrillary acidic protein astrocytopathy presenting as an isolated spinal cord lesion

**DOI:** 10.1097/MD.0000000000036359

**Published:** 2023-11-24

**Authors:** Qing Liu, Hongmei Ma, Dan Yang, Tingting Bian, Jinyuan Ji, Huijie Duan, Heli Yan, Xiangbo Wang

**Affiliations:** a Department of Neurology, Beijing Fengtai You’anmen Hospital, Beijing, China; b Department of Neurology, Xuanwu Hospital, Capital Medical University, Beijing, China.

**Keywords:** antibody, astrocytopathy, glial fibrillary acidic protein, spinal cord

## Abstract

**Introduction:**

Autoimmune glial fibrillary acidic protein astrocytopathy (GFAP-A) is a group of neurological syndromes involving the meninges, brain, spinal cord, and optic nerves and is characterized by sensitivity to steroid therapy. Due to the diverse clinical presentation and lack of uniform diagnostic criteria, GFAP-A can easily be overlooked or diagnosed as another disease. It is even rarer when presenting as an isolated spinal cord lesion.

**Case Report:**

We report the case of a 70-year-old man with initial symptoms of numbness and weakness in both lower limbs, followed by difficulty in urination and defecation, and progression of numbness upward to the hands. Magnetic resonance imaging (MRI) showed a lesion in the spinal cord from cervical level 2 to thoracic 7 in a T2-weighted image. T1-weighted image showed a punctate, lamellar strengthening lesion with significant spinal strengthening. GFAP immunoglobulin G (IgG) was detected in the cerebrospinal fluid and blood. After treatment with intravenous gamma globulin (IVIG), the patient symptoms improved and spinal cord enhancement was reduced.

**Conclusion:**

Long segment cases with punctate and patchy enhancement of the spinal cord are difficult to distinguish from CLAPPERS, so GFAP-A antibody detection is very important. This atypical case also increases neurologists’ understanding of GFAP-A.

## 1. Introduction

GFAP-A was first identified and reported in 2016 by the team to which Dr Boyan Fang belongs with the support of Professor Lennon at the Mayo Clinic.^[1]^ It is a treatable autoimmune inflammatory disease of the central nervous system, with involvement of the meninges, brain, spinal cord, and optic nerve as the primary manifestations, and is sensitive to steroid hormone therapy. A positive GFAP-IgG cerebrospinal test has greater clinical significance than a positive serum test for diagnosing GFAP-A, both in terms of sensitivity and specificity. The currently recognized characteristic imaging presentation of the disease is linear perivascular enhancement perpendicular to the lateral ventricles.^[1–3]^ Isolated myelitis is present in only 5% of patients with GFAP-A^[2,4]^ and is rarely reported nationally or internationally. Herein, we report a case of GFAP-A with isolated myelopathy.

## 2. Case report

**Figure 1.**
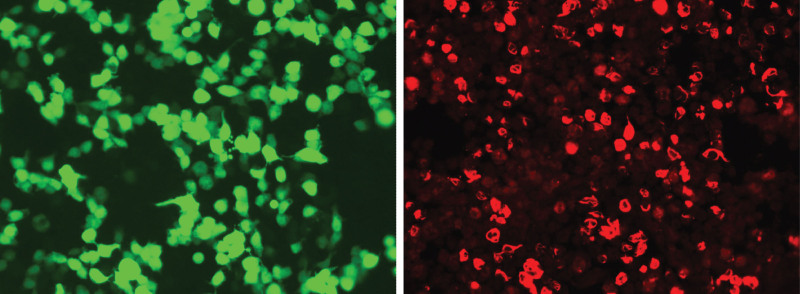
Application of the cytometric bead array (CBA) method to detect GFAP-IgG in the patient cerebrospinal fluid with a titer of 1:100. GFAP = glial fibrillary acidic protein, IgG = immunoglobulin G.

**Figure 2.**
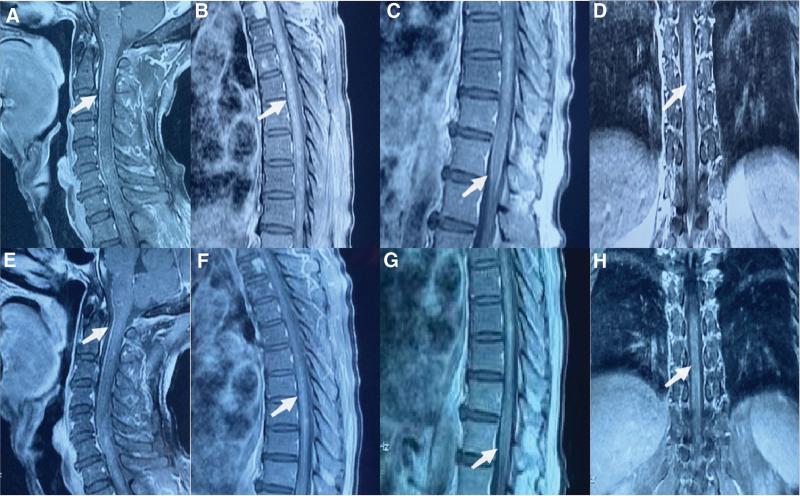
Brain and spinal cord magnetic resonance imaging (MRI) performed before systemic treatment (A, B, C, D): Spinal cord strengthening MRI suggests spinal meninges strengthening (A), cervical and thoracic medullary punctate and lamellar strengthening (B, D) and cauda equina semicircular strengthening (C). Brain and spinal cord magnetic resonance imaging (MRI) performed after systemic treatment (E, F, G, H): spinal cord and spinal meninges strengthening reduced compared to pretreatment.

A 70-year-old male who presented with numbness in both toes for more than 7 months before admission. The numbness gradually progressed upward to the mid-calf after 1 month and was accompanied by difficulty passing urine. Three months later, the patient developed weakness in both lower limbs, which manifested as dragging while walking. After another month, he was unable to walk or squat, and the numbness progressed to his hands, along with urinary retention. Abnormal signals in the spinal cord at the cervical 2–thoracic 7 levels were observed on T1-weighted images. He was diagnosed with “subacute combined degeneration of the spinal cord” by the local hospital and was administered intramuscular vitamins B1 and B12 for approximately 2 weeks. The patient lost 30 kg of body weight within 7 months. He had a previous history of chronic atrophic gastritis with erosion and hypertension, but no family history. Neurological examination revealed decreased near memory and calculation power, grade 2 muscle strength in both lower limbs, positive bilateral Babinski sign, decreased pinprick sensation below the T4 level, and normal deep sensation. Routine blood work suggested elevated neutrophil percentage (78.5%; normal range 42.9%–74.3%), elevated urine routine leukocytes (219/µL normal range 0–5/µL), elevated coagulation D dimer (4.3 mg/L; normal range 0–0.55 mg/L), elevated tumor marker CA72-4 (25.7 U/mL; normal range 0–10 U/mL), positive internal factor antibodies, normal liver and kidney function, syphilis, hepatitis B, HIV-related tests, thyroid function, antinuclear antibody profile, and antineutrophil cytoplasmic antibodies. Lumbar puncture pressure 80 mmH2O, elevated CSF total leukocyte count (26 mm^3^; normal range 0–10 mm^3^), 90% monocytes, elevated CSF protein (77 mg/dL; normal range 8–43 mg/dL), decreased chloride (114.4 mmol/L; normal range 115–130 mmol/L), normal glucose 3.27 mmol/L (concurrent venous glucose 5.4 mmol/L), elevated immunoglobulin A (0.96 mg/dL; normal range 0–0.2 mg/dL), elevated immunoglobulin M (0.36 mg/dL; normal range 0–0.2 mg/dL), elevated immunoglobulin G (IgG) (10.6 mg/dL, normal range mg/dL), positive cerebrospinal fluid GFAP-IgG at a titer of 1:100 (Fig. 1), positive blood GFAP-IgG at a titer of 1:32, weakly positive serum and cerebrospinal fluid IgG oligoclonal bands, positive activated lymphocytes and plasma cells in cytology, no tumor cells were found, antibodies to aquaporin 4 (AQP4), myelin oligodendrocyte glycoprotein (MOG) No abnormalities were found in AQP4 antibody, MOG antibody, paraneoplastic syndrome-related antibody, autoimmune encephalitis-related antibody, tuberculosis antibody, adenosine deaminase, lymphocyte culture + interferon (TB-spot). Enhanced T1-weighted images of the spinal cord suggested punctate and patchy enhancement within the spinal cord at the level of the spinal cord, cervical 2–thoracic 7 (Fig. 2). Abdominopelvic ultrasonography suggested bilateral renal cysts and prostatic hypertrophy with echogenic heterogeneity. Ultrasonography of the deep veins in the lower limbs was suggestive. Electrocardiography revealed sinus tachycardia with T-wave changes. PET-CT suggested: diffuse increased metabolic activity in the cerebral bridge, medulla oblongata, cervical medulla, and upper thoracic medulla; possible inflammatory changes; and hypermetabolic lymph nodes in the mediastinum and bilateral hilar lungs; reactive hyperplasia; Multiple small cysts in the liver, possible left adrenal adenoma, enlarged prostate, slightly hypermetabolic foci in the lower lateral part, and possible inflammation. On admission, the patient was hypothermic, with a temperature of 37.3°C to 37.7°C. After the proposed diagnosis of GFAP-A, we administered intravenous methylprednisolone 250 mg/d for 3 days and 160 mg/d for 3 days. The patient developed hyperthermia at a temperature of 38.5°C, and the hormone was discontinued as the urinary tract infection worsened on laboratory tests and was combined with a bloodstream infection. Intravenous gamma globulin (IVIG) 0.4 g/kg was then applied for 5 days along with moxifloxacin for anti-infection. After treatment, the patient infection was controlled and limb weakness improved. After 20 days of treatment, the T1-weighted image was repeated to show a reduction in the extent of the lesion (Fig. 2), the CSF GFAP-IgG 1:100 was repeated, and the serum GFAP-IgG was negative. One month later, the patient was fever-free and the muscle strength of both lower limbs was grade 4. One month after discharge, the patient limb weakness worsened, and was treated with Chinese herbal medicine at a local hospital. Two weeks later, the patient symptoms improved.

During the 1-year follow-up period, the patient was able to stand with external force, but still left with numbness and pain in their lower limbs. The pain affected sleep and daily life, and he took long-term oral painkillers to control it. Unfortunately, the patient has not been able to undergo a face-to-face consultation, only his family members have described his symptoms over the phone.

## 3. Discussion

GFAP-A is a treatable autoimmune disease of the central nervous system sensitive to steroid hormone therapy. GFAP-A lesions often involve one or more parts of the central nervus system, such as the meninges, brain, spinal cord, and optic nerve, and only 5% of patients with GFAP-A develop isolated spinal cord lesions.^[2,4]^ However, the pathogenesis of GFAP-A remains unclear. Infection may be a common trigger of GFAP autoimmunity in humans, with 2 cases currently reported in China, with evidence of herpes simplex virus infection before onset.^[3,5]^ Other components of the immune system (microglia, macrophages, cytokines, and chemokines) may also contribute to pathogenesis.^[2]^ GFAP is an intracellular antigen, so it is difficult for antibodies to interact with it; therefore, it is generally considered nonpathogenic. However, GFAP can activate T cell-mediated cytotoxic immune responses and cause diseases.^[2]^ It usually has acute or subacute onset and presents with a progressive worsening or relapsing remission course. Clinical manifestations include fever, headache, involuntary movements, myelitis, optic nerve abnormalities, ataxia, psychiatric abnormalities, epilepsy, and other nonspecific signs and symptoms.^[2,4]^ We report the case of an elderly patient who presented with numbness and weakness of both lower limbs and dyscalculia, suggesting that the lesion was located in the spinal cord. The patient was hospitalized and found to have a fever; none of these presentations were clinically specific.

MRI is the test of choice to confirm the diagnosis of GFAP-A, with enhanced lesions visible in 2/3 of the patients.^[2]^ The currently recognized characteristic imaging presentation of the disease is linear perivascular enhancement perpendicular to the lateral ventricles.^[1–3]^ Other MRI enhancement findings include soft meningeal enhancement, punctate enhancement, meander-like (serpentine) enhancement, and periventricular canal enhancement.^[2,3]^ Spinal cord involvement is often a long segmental (≥3 vertebral segments) lesion on MRI. Still the visualization is relatively blurred and less pronounced than that in AQP4-positive and MOG-positive myelitis, with poorly defined borders and more minor spinal cord swelling.^[2,6,7]^ Dotted or linearly enhancing lesions adjacent to GFAP-enriched areas of the central canal of the spinal cord are sometimes observed, and there may also be soft spinal membrane enhancement.^[2,3,7]^ Based on meningitis and perivascular inflammatory pathology, lesion enhancement is the result of contrast leakage from a compromised blood-brain barrier, which is repaired after treatment, and the lesion may disappear.^[2]^ The patient did not have a typical linear lesion perpendicular to the ventricles, which corresponds to his lack of symptoms of neurological damage to the brain. The patient spinal MRI T2-weighted images and strengthening MRI suggested a lesion located throughout the spinal cord with punctate and lamellar strengthening, somewhat similar to the “peppercorn sign” of CLIPPERS. We considered the possibility of a diagnosis of CLIPPERS. Still, the patient had no corresponding signs of brainstem or cerebellar involvement, and we subsequently found positive cerebrospinal fluid GFAP-IgG, which ruled out this diagnosis. The MRI also revealed significant spinal strengthening and cauda equina semicircular strengthening, which is not consistent with the features of CLIPPERS syndrome, but is compatible with the previously reported imaging features of GFAP-A spinal cord lesions, which are much rarer than the typical linear strengthening of the brain, with only one reported case of an adult patient with GFAP-A with superficial spinal cord lesions.^[8]^ Flanagan report of 102 in a study of cerebrospinal fluid GFAP IgG-positive patients showed that the most common clinical presentation was meningoencephalitis.^[3]^ Our patient underwent a post-treatment review of the spinal cord MRI, which revealed fewer foci of enhancement than before, consistent with the recovery of the patient clinical symptoms, which is also compatible with the changes in GFAP-A imaging.

In addition to characteristic imaging findings, positive cerebrospinal fluid GFAP-IgG is also a diagnostic condition for GFAP-A, provided that other diseases that may present similarly, such as various types of meningoencephalitis and neuromyelitis optica spectrum disorders, have been excluded^[1,9]^ and that the rate and titer of antibody positivity in GFAP-A cerebrospinal fluid are significantly higher than in serum.^[1,3]^ The first lumbar puncture of cerebrospinal fluid and blood in our confirmed patients showed positive GFAPIgG and higher cerebrospinal fluid titers than serum, consistent with reports that serum antibody titers turned negative after treatment, with no significant change in cerebrospinal fluid antibody titers, and the decrease in antibody titers matched the improvement in the patients’ clinical symptoms.

Approximately 20% of patients with GFAP-A also have autoimmune diseases such as type 1 diabetes, autoimmune thyroid disease, and rheumatoid arthritis.^[4]^ Some patients may have a combination of other positive antibodies, most commonly NMDA, AQP4, and MOG, and we have reported patients who did not show positive results for these antibodies. However, our patient had a combination of positive endogenous factor antibodies, which are mainly antibodies associated with autoimmune gastritis and autoimmunity. This test is rarely performed because most patients with GFAP-A do not have gastric symptoms. Tumors are detected in approximately 25% of patients about 2 years after presentation, and the most common tumor is ovarian teratoma (75%), usually a mature teratoma, with other cancers being rare and diverse.^[3,10]^ Our patient had no tumors detected at this time after whole-body PET/CT screening, and regular tumor-related investigations have been recommended.

Treatment of GFAP-A in the acute phase consists of high-dose steroid hormone shock therapy; usually, methylprednisolone 1000 mg/d is shared for 3 to 5 days and then switched to oralor IVIG and plasma exchange, with 70% of patients responding well to hormones.^[6]^ Most patients have a monophasic course, but approximately 20% to 50% relapse with hormone tapering, with relapse requiring re-shock and long-term immune maintenance therapy.^[2]^ Our patient was older and had gastric erosions and a urinary tract infection; therefore, high-dose steroid shocks were not chosen initially, and only moderate doses were administered. However, the hormones were subsequently discontinued due to worsening illness, disease, condition, and IVIG administration. This is the biggest challenge encountered in this study. The most effective treatment for this disease is steroids, but the patient had to change treatment due to worsening urinary tract infections. This may be one of the reasons for the poor prognosis of the patient.

The re-exacerbation of the patient symptoms after 1 month is thought to be related to the metabolism of IVIG, a rapid immunomodulatory drug, and the patient should receive long-term immunomodulatory drug maintenance therapy after the exacerbation. Long-term follow-up of 7 Chinese patients in one study showed that severe disability and poor outcomes were the norms, possibly due to late diagnosis (median duration of symptoms at diagnosis was 12 months) or more severe disease in Chinese patients.^[11]^ Our patient was diagnosed after 8 months and did not fully recover after the 1st treatment and still had some residual neurological deficits, probably due to late therapeutic intervention. The inability to use long-term immunomodulatory maintenance therapy after relapse due to recurrent infections also contributed to the patient poor recovery.

In conclusion, clinicians are increasingly recognizing GFAP-A. If a patient is clinically found to have positive cerebrospinal fluid GFAP or typical imaging findings, GFAP-A should be considered when clinical symptoms, signs, and ancillary findings cannot be explained by other diseases, and early diagnosis and immunotherapeutic intervention is an effective way to improve the prognosis of patients with GFAP-A.

## Author contributions

**Conceptualization:** Qing Liu.

**Data curation:** Qing Liu, Dan Yang.

**Formal analysis:** Qing Liu, Xiangbo Wang.

**Investigation:** Tingting Bian.

**Software:** Hongmei Ma, Jinyuan Ji.

**Supervision:** Qing Liu, Heli Yan.

**Visualization:** Huijie Duan.

**Writing – original draft:** Qing Liu.

**Writing – review & editing:** Qing Liu.
